# Association between Early Anatomic Response and Intraocular Pressure Change after Intravitreal Dexamethasone Implant: An Optical Coherence Tomography Study

**DOI:** 10.3390/jcm9092692

**Published:** 2020-08-20

**Authors:** Kyung Tae Kim, Hwanho Lee, Jin Young Kim, Ju Byung Chae, Sungmin Hyung, Dong Yoon Kim

**Affiliations:** 1 Department of Ophthalmology, Gangneung Asan Hospital, University of Ulsan College of Medicine, Gangneung 25440, Korea; kkt400@gmail.com; 2 Department of Ophthalmology, Chungbuk National University Hospital, College of Medicine, Chungbuk National University, Cheongju 28644, Korea; brolaril@naver.com (H.L.); cjbmed@naver.com (J.B.C.); smh@chungbuk.ac.kr (S.H.); 3 Department of Ophthalmology, Jeju National University Hospital, Jeju National University School of Medicine, Jeju 63241, Korea; umuse7@gmail.com

**Keywords:** dexamethasone implant, macular edema, intraocular pressure, central subfield thickness

## Abstract

Purpose: To investigate the associations between early anatomical responses and intraocular pressure (IOP) changes in macular edema (ME) due to retinal vascular diseases treated with an intravitreal dexamethasone (DEX) implant. Methods: A retrospective review was conducted involving ME patients who underwent intravitreal DEX implantation. The eyes were divided into increased IOP (IIOP) or non-IIOP (nIIOP) groups according to the presence or absence of significant IOP elevation. Significant IOP elevation was defined by both the absolute value of IOP elevation (5 mmHg or higher) and an elevation percentage of the baseline IOP (an increase equal to 30% of the pre-injection IOP or higher). We analyzed the difference in central subfield thickness (CST) change according to the IOP elevation after DEX implantation. Relationships between IOP change and CST reduction after intravitreal DEX implantation were analyzed by Pearson correlation coefficients. Results: A total of 49 eyes, 29 with diabetic ME and 20 with ME due to retinal vein occlusion (RVO), were included in this study. Of the 49 eyes, 18 eyes (36.7%) were classified as IIOP group and 31 (63.3%) as nIIOP group. Significant differences in mean CST reductions over baseline one week after DEX implantation were observed between the groups. The degree of CST reduction from baseline to 1 week was significantly correlated with the degree of IOP change from baseline at 1 week and 1 month after intravitreal DEX implantation. Conclusions: In patients with ME due to retinal vascular diseases, we noted an early anatomical response significantly correlated with IOP change after intravitreal DEX implantation. Therefore, patients with favorable early anatomical responses to DEX implantation should be carefully monitored for IOP elevation.

## 1. Introduction

The intravitreal dexamethasone (DEX) implant (Ozurdex, Allergan, Inc., Irvine, CA, USA) is a sustained-release device that is approved for the treatment of macular edema associated with diabetic retinopathy, retinal vein occlusions and noninfectious posterior uveitis [1,2,3,4,5,6].

Despite its efficacy, elevations in intraocular pressure (IOP) and cataract formation are well-known side effects of the DEX implant [7,8]. IOP increases due to the steroid can be attributed to the class effect related to the intrinsic activity of steroids within the trabecular meshwork, such as microstructural changes or the deposition of extracellular matrix (ECM) [8,9]. In the three-year, randomized, sham-controlled trial of dexamethasone intravitreal implant in patients with Diabetic Macular Edema (MEAD trial), 32% of eyes had IOP above 25 mmHg and 6.6% of eyes had IOP above 35 mmHg after DEX implantation [6]. In addition, 41.5% of eyes required IOP-lowering medication, but only 0.6% of eyes underwent surgical procedures, such as trabeculectomy, within 3 years [6]. Maturi et al. [8] reported that IOP increases greater than 10 mmHg from baseline after the DEX implant occurred in 27.7% of patients with diabetic macular edema (DME), and their frequency did not increase with repeated injections. Clinically significant increases in IOP were reported in approximately one-third of patients treated with the DEX implant, and increases in IOP were typically controlled with medication and rarely required control via surgical procedure [6].

Chae et al. [10] reported that following the injection of intravitreal triamcinolone acetonide (IVTA) to treat branch retinal vein occlusion (BRVO), steroid responders (defined as IOP > 22 mmHg) experienced a greater reduction in macular edema (ME) than non-responders. Another study also reported that the mean average change in central subfield thickness (CST) from baseline after DEX implantation was higher in patients with an IOP increase of more than 10 mmHg than it was in patients without [8]. These results suggest a correlation between the degree of anatomic improvement and IOP elevation after intravitreal steroid injection in ME. That is, a favorable initial anatomic response after DEX implantation might increase the risk of IOP elevation, and IOP-lowering medication could be used prophylactically to prevent this IOP elevation. Therefore, the purpose of this study was to investigate whether IOP elevation could be predicted by analyzing the degree of early CST reduction via spectral-domain optical coherence tomography (SD-OCT) after intravitreal DEX implantation for DME and ME due to retinal vein occlusion (RVO). 

## 2. Methods

A retrospective review was conducted involving patients who received intravitreal DEX implantation for the management of ME due to retinal vascular diseases, such as diabetic retinopathy (DR) or RVO, at the Chungbuk National University Hospital in Korea between March 2016 and June 2018. The main purpose of this study was to analyze the association between early CST reduction and IOP change after intravitreal DEX implantation, and to investigate the possibility of predicting IOP elevation by analyzing early CST changes via SD-OCT after intravitreal DEX implantation. The approval of the Institutional Review Board and ethics committees of Chungbuk National University Hospital was granted before the initiation of the study, which was performed in compliance with the tenets of the Declaration of Helsinki. Because of the retrospective study design, this research involved no more than minimal risk to the subjects. Therefore, the IRB gave exemption from the requirement of obtaining informed consent.

### 2.1. Inclusion and Exclusion Criteria

The inclusion criteria were patients treated with intravitreal DEX implantation for DME or ME due to RVO. Intravitreal DEX implantation was performed when the CST was over 300 μm in SD-OCT. The exclusion criteria were the following: (1) another concomitant ocular disease that could cause macular edema (i.e., neovascular age-related macular degeneration or choroidal neovascularization due to other reasons, uveitis, or recent intraocular surgery possibly causing postsurgical macular edema); (2) intravitreal anti-vascular endothelial growth factor or any kinds of steroid injection in the three months preceding the dexamethasone injection; (3) history of panretinal photocoagulation in the three months preceding the dexamethasone injection; (4) another ocular condition that compromises visual acuity other than cataract; (5) glaucoma referral, history of steroid-induced IOP increase or ocular hypertension characterized by IOP increases over 21 mmHg without anti-glaucoma medication in the study eye; (6) current use of anti-glaucoma medication. We consecutively included patients who met the inclusion and exclusion criteria between March 2016 and June 2018.

### 2.2. Intravitreal DEX Implantation

The DEX implant was inserted into the vitreous cavity through the pars plana using a customized, single-use, 22-gauge applicator. The eyes were anesthetized with topical anesthetics before intravitreal DEX implantation, and treated with a topical moxifloxacin (Vigamox^®^, Alcon Laboratories, Ft. Worth, TX, USA) 4 times daily for 7 days after the procedure.

### 2.3. Outcome Measurements

Best corrected visual acuity (BCVA) tests using the Snellen chart, IOP measurement, slit-lamp examination, fundus photography, fluorescein angiography and SD-OCT (Spectralis; Heidelberg Engineering, Heidelberg, Germany) were performed on all patients at the initial visit. At each subsequent visit, patients underwent assessment of BCVA, IOP measurement, slit-lamp examination, dilated fundus examination, fundus photography and SD-OCT. IOP was measured by one ophthalmologist (HL). IOP was measured in mmHg with the same Goldmann applanation tonometry at each time point. Eyes were treated with topical anti-glaucoma medication if presenting with an IOP over 21 mmHg. For SD-OCT images, the volumetric scans of Spectralis SD-OCT were acquired with the Spectralis Viewing Module (Version 6.0.9.0). A custom 20° × 20° volume acquisition protocol, which covered the 6 mm × 6 mm surface of the macula, was used to obtain one set of high-speed scans from each eye. With this protocol, 49 cross-sectional B-scan images were obtained, each composed of 512 A-scans [11]. The integrated follow-up mode of the device was used to ensure that the exact same retinal area was imaged at every follow-up visit. The quantitative assessment of ME included CST, which was calculated automatically by the instrument. These data were collected at baseline and at 1 week, 1 month and 3 months after intravitreal DEX implantation. 

### 2.4. Association between IOP Change and CST Change after DEX Implantation

First of all, the eyes were divided into two groups according to IOP change at 1 week after intravitreal DEX implantation in order to compare the degree of CST change as it related to IOP change. Eyes showing significant increases in IOP at 1 week compared with pretreatment IOP levels were categorized into the increase of IOP (IIOP) group, and the others were categorized into the non-IIOP (nIIOP) group. Significant IOP elevation was defined both by the absolute value of IOP elevation (5 mmHg or higher) and an elevation percentage of baseline IOP (an increase equal to 30% of the pre-injection IOP or higher) [12]. 

To know whether the different amount of CST reduction after DEX implantation was related to IOP elevation, we also divided the included eyes into two groups according to CST change at 1 week after intravitreal DEX implantation. Eyes with over 25% reduction in CST at 1 week after intravitreal DEX implantation were categorized into the good early anatomic response (GEAR) group, and the others were categorized into the poor early anatomic response (PEAR) group [13]. The BCVA, IOP and CST values at baseline, 1 week, 1 month and 3 months were compared between these two groups.

### 2.5. Statistical Analysis

SPSS version 22.0 software (SPSS, Inc., Chicago, IL, USA) was used to perform the statistical analyses, and a *p* < 0.05 was considered statistically significant. All values are presented means ± SD or numbers (%). The assessment of normality was conducted using the Kolmogorov–Smirnov test. 

Differences in parameters, including BCVA, IOP and CST, between groups were evaluated using the Student’s t-test. Comparisons between categorical variables were performed with the Pearson’s χ^2^ test. Pearson correlation coefficients (*r*) were calculated and used to elucidate relationships between IOP change and CST reduction after intravitreal DEX implantation.

## 3. Results

### 3.1. Baseline Characteristics

The patients’ demographics and baseline ocular findings are summarized in Table 1. A total of 29 eyes from 29 patients with DME (14 eyes with nonproliferative diabetic retinopathy (NPDR), 15 eyes with proliferative diabetic retinopathy (PDR)) and 20 eyes from 20 patients with ME due to RVO (14 eyes with BRVO, 6 eyes with central retinal vein occlusion (CRVO)) were included in this study. The mean age of the patients was 55.1 ± 10.4 years. In total, 25 eyes (51%) had undergone panretinal photocoagulation, and none of the eyes had undergone macular laser photocoagulation before intravitreal DEX implantation. The mean number of prior intravitreal bevacizumab injections was 3.3 ± 3.4. The mean baseline BCVA was 0.63 ± 0.48 logMAR units, and the mean baseline CST was 538.9 ± 105.3 μm.

### 3.2. Degree of CST Reduction According to IOP Change 

The eyes were divided into two groups according to the presence or absence of significant IOP elevation at 1 week after intravitreal DEX implantation. Of the 49 eyes, 18 eyes (36.7%) were classified into the IIOP group and 31 eyes (63.3%) into nIIOP group. There were no statistically significant differences in initial BCVA, IOP or CST between the two groups. 

**Figure 1:**
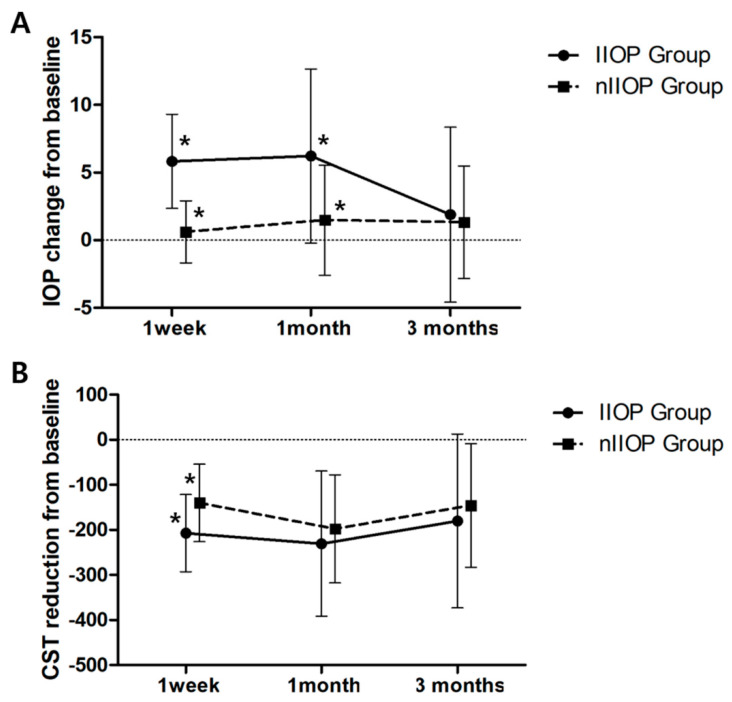
Graph illustrating changes in mean intraocular pressure (IOP) elevation and mean central subfield thickness (CST) reduction after intravitreal dexamethasone (DEX) implantation between the increase of IOP (IIOP) group and non-IIOP (nIIOP) group. (**A**) Mean IOP change. (**B**) Mean CST reduction. Asterisk (*) means significant difference between the groups.

The mean IOP changes and CST reductions after intravitreal DEX implantation of the IIOP and nIIOP groups are shown in Figure 1. The mean IOP changes in the IIOP group at 1 week, 1 month and 3 months after intravitreal DEX implantation were 5.83 ± 3.47, 6.22 ± 6.43 and 1.89 ± 6.46, respectively. In the nIIOP group, the mean IOP changes at 1 week, 1 month and 3 months after intravitreal DEX implantation were 0.61 ± 2.29, 1.48 ± 4.07 and 1.32 ± 4.15, respectively. The mean IOP changes at 1 week and 1 month between the groups were significantly different (*p* < 0.001 at 1 week, *p* = 0.003 at 1 month). The mean CST in the IIOP group was higher than that in the nIIOP group at baseline, but there was no significant difference (574.44 ± 109.53 in IIOP group, 518.23 ± 98.67 in nIIOP group, *p* = 0.071). In the IIOP group, the mean CST reductions at 1 week, 1 month and 3 months after intravitreal DEX implantation were −207.11 ± 85.48, −230.67 ± 161.20 and −180.06 ± 192.64, respectively. In the nIIOP group, the mean CST reductions at 1 week, 1 month and 3 months after intravitreal DEX implantation were −140.29 ± 86.19, −197.71 ± 119.86 and −145.55 ± 136.98, respectively. Significant difference was observed between the groups at 1 week after DEX implantation (*p* = 0.012).

The number of patients treated with IOP-lowering medication at 1 week, 1 month and 3 months were significantly different between the two groups (Table 2). Nine eyes (50.0%) were treated with topical anti-glaucoma medication at 1 week after DEX implant, and 11 eyes (61.1%) were thus treated at 1 month and 3 months in the IIOP group. In contrast to the IIOP group, no patients from the nIIOP group were treated with anti-glaucoma medication at 1 week after DEX implantation. Only four eyes (12.9%) were treated with topical antiglaucoma medication at 1 month, and six eyes (19.4%) at 3 months after DEX implantation. 

### 3.3. Degree of IOP Elevation According to CST Change 

**Figure 2:**
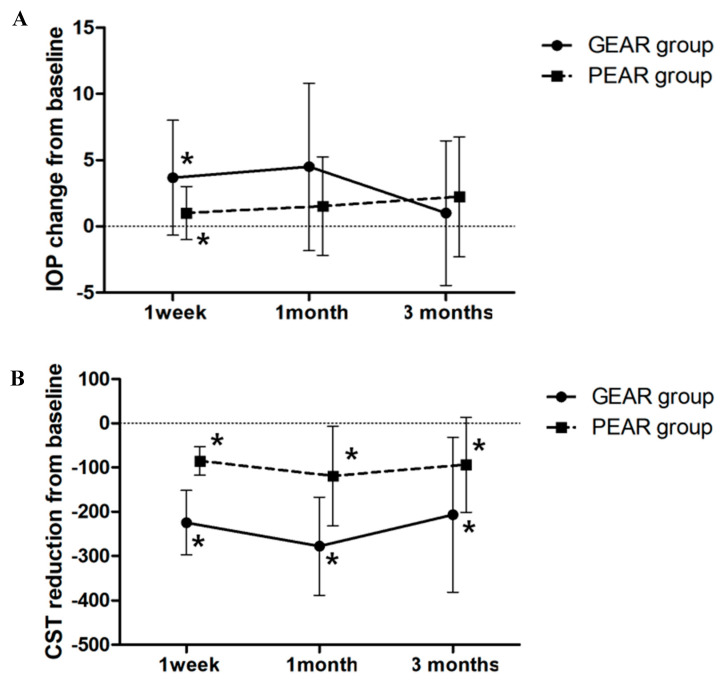
Graph illustrating changes in mean intraocular pressure (IOP) elevation and mean central subfield thickness (CST) reduction after intravitreal dexamethasone (DEX) implantation between good early anatomic responder (GEAR) and poor early anatomic responder (PEAR) groups. (**A**) Mean IOP change. (**B**) Mean CST reduction. Asterisk (*) means significant difference between the groups.

The eyes were divided into GEAR and PEAR groups according to the degree of CST reduction rate at 1 week after intravitreal DEX implantation. Of the 49 eyes, 28 eyes (57.1%) were classified into the GEAR group, and 21 eyes (42.9%) were classified into the PEAR group. The GEAR group included 7 eyes with severe NPDR, 8 eyes with PDR, 11 eyes with BRVO and 2 eyes with CRVO, and the PEAR group included 7 eyes with severe NDPR, 7 eyes with PDR, 3 eyes with BRVO and 4 eyes with CRVO. The mean IOP changes and CST reductions from baseline after the intravitreal DEX implantation of the GEAR and PEAR groups are shown in Figure 2. The mean IOP changes in the GEAR group at 1 week, 1 month and 3 months after intravitreal DEX implantation were 3.68 ± 4.33, 4.50 ± 6.31 and 1.00 ± 5.45, respectively. In the PEAR group, the mean IOP changes at 1 week, 1 month and 3 months after intravitreal DEX implantation were 1.00 ± 2.00, 1.52 ± 3.71 and 2.24 ± 4.53, respectively. The mean IOP changes at 1 week were significantly different between the groups (*p* = 0.006). The mean CST reductions in the GEAR group at 1 week, 1 month and 3 months after intravitreal DEX implantation were −224.68 ± 72.75, −277.79 ± 110.45 and −206.57 ± 174.70, respectively. In the PEAR group, the mean CST reductions at 1 week, 1 month and 3 months after intravitreal DEX implantation were −85.05 ± 32.43, −119.19 ± 112.67 and −93.76 ± 107.38, respectively. Significant differences between groups were observed at 1 week, 1 month and 3 months after DEX implantation (*p* < 0.001 at 1 week and 1 month, *p* = 0.012 at 3 months). 

**Figure 3:**
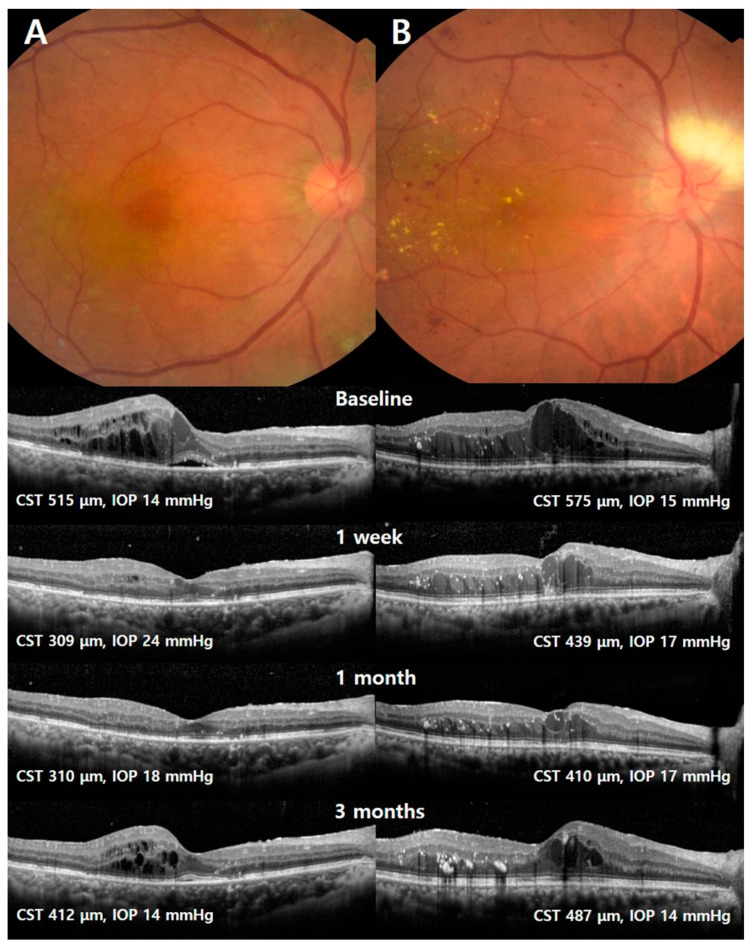
Fundus photographs and Optical coherence tomography (OCT) scans of representative cases from the good early anatomic responder (GEAR) and poor early anatomic responder (PEAR) groups. The left column (**A**) represents the GEAR group, and the right column (**B**) represents the PEAR group.

Representative cases from each of the two groups are shown in Figure 3. The first case (left column) was a 58-year-old female with severe NPDR, and the second case (right column) was a 63-year-old female with PDR. Each eye received two rounds of intravitreal bevacizumab injection and panretinal photocoagulation (PRP) 3 months before intravitreal DEX implantation. The Snellen best-corrected visual acuity (BCVA) was 0.4, the central subfield thickness (CST) was 515 μm, and the intraocular pressure (IOP) was 14 mmHg at baseline in the eye of the GEAR group. At 1 week after intravitreal DEX implantation, the CST decreased to 309 μm and the IOP increased to 24 mmHg, and the patient was treated with topical anti-glaucoma agents. In the case of the PEAR group, the Snellen BCVA was 0.3, the CST was 575 μm and the IOP was 15 mmHg at baseline. At 1 week after intravitreal DEX implantation, the CST decreased to 439 μm and the IOP increased to 17 mmHg. At 3 months, the CST increased to 487 μm, but the IOP was stable at 14 mm.

### 3.4. Correlation between IOP Change and CST Reduction

**Figure 4:**
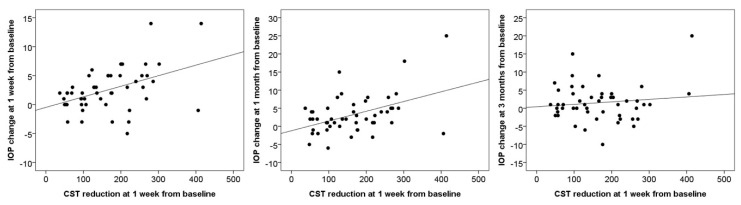
Scattergrams of intraocular pressure (IOP) changes versus central subfield thickness (CST) reductions. The amount of CST reduction at 1 week was significantly correlated with the degree of IOP change at 1 week and 1 month after intravitreal DEX implantation (*r* = 0.443, *p* = 0.001; *r* = 0.445, *p* = 0.001, respectively). There was no significant correlation between the amount of CST reduction at 1 week and the degree of IOP change at 3 months (*r* = 0.122, *p* = 0.404).

The potential relationships between IOP change and anatomical response were analyzed using Pearson’s correlation coefficients. The degree of CST reduction at 1 week was significantly correlated with the degree of IOP change at 1 week and at 1 month after intravitreal DEX implantation (*r* = 0.443, *p* = 0.001; *r* = 0.122, *p* = 0.001, respectively). However, no significant correlation was observed between the amount of CST reduction at 1 week and the amount of IOP change at 3 months (*r* = 0.122, *p* = 0.404) (Figure 4). There was one patient with very severe changes in IOP (more than 14 mmHg) and CST (more than 400 μm) after dexamethasone implantation. When we excluded that patient, the degree of CST reduction at 1 week was also significantly correlated with the degree of IOP change at 1 week and 1 month after intravitreal DEX implantation (*r* = 0.323, *p* = 0.025; *r* = 0.286, *p* = 0.048, respectively), and was not correlated with the degree of IOP change at 3 months (*r* = −0.116, *p* = 0.431).

## 4. Discussion

In this study, we investigated the association between IOP increase and CST reduction after intravitreal DEX implantation. The primary finding was that the degree of CST reduction in the IIOP group at 1 week was greater than that in the nIIOP group (−207.11 ± 85.48 in IIOP group, −140.29 ± 86.19 in nIIOP group, *p* = 0.012). Similarly, the degree of IOP elevation at 1 week in the GEAR group was greater than that in the PEAR group (3.68 ± 4.33 in GEAR group, 1.00 ± 2.00 in PEAR group, *p* = 0.006). In addition, the amount of CST reduction at 1 week was significantly correlated with the degree of IOP change both at 1 week and 1 month (*r* = 0.443 at 1 week, *p* = 0.001; *r* = 0.122 at 1 month, *p* = 0.001).

Chae at al. [10] reported that steroid responders, defined as eyes with an IOP greater than 22 mmHg at 1 month after IVTA, experienced a greater overall reduction in macular edema than non-responders at the 1-month follow-up. Another study also reported that the average reduction in CST was numerically larger in patients who had an IOP increase of at least 10 mmHg after DEX implant treatment compared to those without such an IOP increase [8]. In our study, similar to previous studies, the CST reduction was greater in the IIOP group than in the nIIOP group. Based on these results, it can be assumed that there is a possibility of association between IOP elevation and anatomical improvement after intravitreal DEX implantation for the treatment of ME. We also found a significant positive correlation between IOP elevation and CST reduction at 1 week after dexamethasone implant. After comparing the degree of IOP elevation between GEAR and PEAR groups according to the degree of CST improvement after intravitreal DEX implantation, we found that the degree of IOP elevation was greater in the GEAR group than in the PEAR group. These results suggest the possibility of predicting which eyes will present with IOP elevation after dexamethasone implantation by evaluating CST decreases at 1 week after dexamethasone injection. This prediction could be of importance for clinical practice, allowing physicians to infer in advance the potential for IOP elevation after intravitreal DEX implantation by measuring the amount of CST reduction at 1 week. Anti-glaucoma medication could then be used prophylactically, to prevent nerve damage caused by IOP elevation.

Steroid-induced IOP increases have been known as a class effect related to the intrinsic activity of steroids within the trabecular meshwork, but the mechanism by which they occur is highly debated [9]. Changes in the microstructure of the trabecular meshwork may cause a decrease in outflow capacity and an increase in IOP [14]. Clark et al. [15] discovered that dexamethasone facilitated the cross-linkage of actin fibers in cultured human trabecular meshwork cells, leading to the formation of networks that decreased aqueous outflow. Corticosteroids also increase the deposition of the extracellular matrix in the trabecular meshwork, which can reduce outflow capacity [16]. Several studies have reported that dexamethasone treatment inhibits arachidonic acid metabolism in the trabecular meshwork cell and reduces the phagocytic properties of the cells [17,18]. A decreased outflow capacity following the reduced degradation of substances in the trabecular meshwork due to steroids may also result in an increase in IOP.

Prostaglandin analogs are among the class of ocular hypotensive drugs with a favorable profile of hypotensive efficacy [19]. Prostaglandins could influence outflow facility by regulating cellular cyclic-AMP levels [20]. Steroid treatment improves macular edema in many ocular diseases by inhibiting inflammatory reaction, but at the same time, inhibiting arachidonic acid metabolism and decreasing prostaglandins secretion can lead to elevations in IOP. Weinreb et al. [17] reported that the marked inhibition of prostaglandin secretion observed following dexamethasone treatment of human trabecular cells might explain the tendency of this steroid to produce elevations in IOP. Prostaglandin might be thought to be a clue to explaining the possibility of the association between IOP change and CST change. We surmise that the degree of response to retinal edema following steroid injection may indirectly indicate the effect of steroids on the trabecular meshwork. Therefore, in instances of favorable anatomical response to the steroid, the IOP may paradoxically rise because of the simultaneous effects of steroids on the trabecular meshwork. In addition to prostaglandin, interleukin 1 (IL-1) might be a clue to explaining the possibility of the association between IOP change and CST change. IL-1 is a proinflammatory cytokine that regulates the expression of a wide variety of target genes and proteins [21]. A study reported that IL-1 stimulates aqueous outflow by directly increasing paracellular permeability across Schlemm’s canal [22,23]. Inhibition of the inflammation by steroids reduces the expression of IL-1, and this could lead to IOP elevation. In ME patients showing greater decreases in IL-1 levels, there was a greater decrease in ME [24], which can conversely lead to a risk of IOP elevation due to the suppression of IL-1. Furthermore, the glucocorticoid receptor could also be a possible reason. The glucocorticoid receptor can exist in multiple isoforms, such as classic cytoplasmic glucocorticoid receptor-α (GRα) or GRβ [23,25]. In the presence of GRα, GRβ functions as a dominant negative inhibitor and antagonizes GRα activity [23,25]. Therefore, differences in the degree of genetic predisposition to the glucocorticoid receptor might be another explanation of the fact that the greater the improvement in CST is, the greater the risk of IOP elevation will become.

In the MEAD study evaluating DEX implantation for the treatment of DME, increases in IOP were most commonly observed at 1.5 or 3 months after intravitreal DEX implantation [6,8]. The Geneva study group’s (2011) results showed that a maximum rise of IOP was observed within 60 days of implantation in patients with ME due to BRVO [1]. Similarly, Chin et al. [26] have reported that the greatest IOP elevation was seen at the 1.5-month to 2.5-month follow-up interval. A study into IOP elevation with intravitreal DEX implantation in the real world reported that the cumulative probability of having an IOP ≥ 21 mmHg was 20% at 1–2 weeks, and the probability of having an IOP ≥ 25 mmHg or ≥35 mmHg was 5% and 2% at 1–2 weeks, respectively [27]. In our study, of the 17 eyes treated with anti-glaucoma medication due to an IOP elevation greater than 21 mmHg over the three-months study period, 9 eyes (52.94%) presented with an IOP increase at 1 week, 6 eyes (35.29%) at 1 month, and 2 eyes (11.77%) at 3 months. The peak of IOP elevation occurred between 1 and 3 months, in accordance with results from previous studies. 

Several studies have shown that in the majority of cases demonstrating a rise in IOP after intravitreal DEX implantation, IOP is brought down to within normal limits by anti-glaucoma medication, with very few requiring surgery [8,28]. One study reported that IOP-lowering medications and laser treatments were sufficient for the treatment of raised IOP after corticosteroids [29]. In this current study, IOP was generally managed with one to two topical medications, and none of the patients needed more than three concomitant IOP-lowering medications at any point during the study. Because IOP elevation can be managed by topical anti-glaucoma medication, predicting future IOP elevation and proactively prescribing eye drops might reduce the optic nerve damage caused by the unrecognized IOP elevation, and safely extend the follow-up interval.

The strength of the current study is that it provides additional insight into our understanding of the association between IOP change and CST change after intravitreal DEX implantation in eyes with ME due to retinal vascular diseases. We also propose a prediction method for determining whether a patient will present with IOP elevation after dexamethasone implantation, as results indicate that those with more favorable early anatomical responses are at a higher risk for IOP elevation. However, our study had some notable limitations that were inherent in its retrospective and nonrandomized design. Second, although the elevation of IOP might be more important for glaucoma patients or steroid responders, we did not include these patients in this study. Third, in the current study, one ME patient with very severe changes in IOP (more than 14 mmHg) and CST (more than 400 μm) after dexamethasone implantation was included. This patient could affect the results of the correlation between the IOP change and the CST reduction. However, even if we excluded this patient with very severe changes in IOP and CST after dexamethasone implantation, we could still get the same study result for the correlation between IOP elevation and the CST reduction. Fourth, the baseline CST of the IIOP group was thicker than that of the nIIOP group. Though this difference between the baseline CSTs of the IIOP and nIIOP groups was not significant, it could be one of the reasons why the CST change 1 week after injection was significantly high. Therefore, to verify our study result, further prospectively designed studies with a larger sample size, including glaucoma patients or steroid responders, are warranted.

In conclusion, this study demonstrated that early anatomical response might correlate with IOP change after intravitreal DEX implantation in patients with ME due to retinal vascular diseases, particularly with regard to the degree of CST reduction at 1 week and the degree of IOP elevation at 1 week or 1 month. Therefore, patients with favorable early anatomical responses to intravitreal DEX implantation should be carefully monitored and considered for prophylactic anti-glaucoma medications in order to mitigate IOP elevation.

## Figures and Tables

**Table 1:** Patient demographics and baseline ocular findings.

Characteristics.	Value
No. of patients	49
No. of eyes	49
Age, years (mean ± SD)	55.1 ± 10.4
Sex, male/female (%)	22/27 (45/55)
Hypertension (%)	16 (33)
No. of prior intravitreal bevacizumab injections (mean ± SD)	3.3 ± 3.4
Panretinal photocoagulation (%)	25 (51)
Best-corrected visual acuity, log MAR (mean ± SD)	0.63 ± 0.48
Intraocular pressure, mmHg (mean ± SD)	14.43 ± 3.01
Central macular thickness, μm (mean ± SD)	538.9 ± 105.3
Diagnoses (*n*)	
NPDR	14
PDR	15
BRVO	14
CRVO	6

NPDR = nonproliferative diabetic retinopathy; PDR = proliferative diabetic retinopathy; BRVO = branch retinal vein occlusion; CRVO = central retinal vein occlusion; logMAR = logarithm of the minimal angle of resolution; SD = standard deviation.

**Table 2:** Comparisons of BCVA, IOP and CST between the IIOP and nIIOP groups over 3 months.

Characteristics.	IIOP (*n* = 18)	nIIOP (*n* = 31)	*p* Value
Age, years (mean ± SD)	51.89 ± 10.40	56.90 ± 10.04	0.103 *
Sex, male/female (%)	11/7 (61/39)	11/20 (35/65)	0.082 ^†^
Baseline			
BCVA, log MAR (mean ± SD)	0.76 ± 0.53	0.56 ± 0.40	0.155 *
IOP, mmHg (mean ± SD)	15.06 ± 3.93	14.07 ± 2.31	0.339 *
CST, μm (mean ± SD)	574.44 ± 109.53	518.23 ± 98.67	0.071 *
No. of patients treated with IOP lowering medication	0	0	
1 week after IVDI			
BCVA, log MAR (mean ± SD)	0.49 ± 0.32	0.44 ± 0.26	0.484 *
IOP, mmHg (mean ± SD)	20.89 ± 3.48	14.68 ± 3.29	<0.001 *
CST, μm (mean ± SD)	367.33 ± 75.13	377.94 ± 56.58	0.578 *
No. of patients treated with IOP lowering medication	9	0	<0.001 ^†^
1 month after IVDI			
BCVA, log MAR (mean ± SD)	0.39 ± 0.33	0.42 ± 0.24	0.657 *
IOP, mmHg (mean ± SD)	21.28 ± 6.52	15.55 ± 4.75	0.001 *
CST, μm (mean ± SD)	343.78 ± 138.71	320.52 ± 74.87	0.448 *
No. of patients treated with IOP lowering medication	11	4	0.001 ^†^
3 months after IVDI			
BCVA, log MAR (mean ± SD)	0.38 ± 0.35	0.53 ± 0.40	0.189 *
IOP, mmHg (mean ± SD)	16.94 ± 5.79	15.39 ± 3.50	0.245 *
CST, μm (mean ± SD)	394.39 ± 156.29	372.68 ± 108.68	0.570 *
No. of patients treated with IOP lowering medication	11	6	0.005 ^†^

BCVA = best-corrected visual acuity; logMAR = logarithm of the minimal angle of resolution; IOP = intraocular pressure; CST = central subfield thickness; IIOP = increased intraocular pressure; nIIOP = non-increased intraocular pressure; IVDI = intravitreal dexamethasone implant; SD = standard deviation. * *p* values for Student’s *t*-test; ^†^ *p* values for Pearson’s chi-square test.

## References

[1] Haller J.A., Bandello F., Belfort R., Blumenkranz M.S., Gillies M., Heier J., Loewenstein A., Yoon Y.H., Jacques M.L., Jiao J. (2010). Randomized, sham-controlled trial of dexamethasone intravitreal implant in patients with macular edema due to retinal vein occlusion. Ophthalmology.

[2] Boyer D.S., Faber D., Gupta S., Patel S.S., Tabandeh H., Li X.Y., Liu C.C., Lou J., Whitcup S.M. (2011). Dexamethasone intravitreal implant for treatment of diabetic macular edema in vitrectomized patients. Retina.

[3] Haller J.A., Bandello F., Belfort R., Blumenkranz M.S., Gillies M., Heier J., Loewenstein A., Yoon Y.H., Jiao J., Li X.Y. (2011). Dexamethasone intravitreal implant in patients with macular edema related to branch or central retinal vein occlusion twelve-month study results. Ophthalmology.

[4] Lowder C., Belfort R., Lightman S., Foster C.S., Robinson M.R., Schiffman R.M., Li X.Y., Cui H., Whitcup S.M. (2011). Dexamethasone intravitreal implant for noninfectious intermediate or posterior uveitis. Arch. Ophthalmol..

[5] Robinson M.R., Whitcup S.M. (2012). Pharmacologic and clinical profile of dexamethasone intravitreal implant. Expert Rev. Clin. Pharmacol..

[6] Boyer D.S., Yoon Y.H., Belfort R., Bandello F., Maturi R.K., Augustin A.J., Li X.Y., Cui H., Hashad Y., Whitcup S.M. (2014). Three-year, randomized, sham-controlled trial of dexamethasone intravitreal implant in patients with diabetic macular edema. Ophthalmology.

[7] Capone A., Singer M.A., Dodwell D.G., Dreyer R.F., Oh K.T., Roth D.B., Walt J.G., Scott L.C., Hollander D.A. (2014). Efficacy and safety of two or more dexamethasone intravitreal implant injections for treatment of macular edema related to retinal vein occlusion (Shasta study). Retina.

[8] Maturi R.K., Pollack A., Uy H.S., Varano M., Gomes A.M., Li X.Y., Cui H., Lou J., Hashad Y., Whitcup S.M. (2016). Intraocular pressure in patients with diabetic macular edema treated with dexamethasone intravitreal implant in the 3-year mead study. Retina.

[9] Thakur A., Kadam R., Kompella U.B. (2011). Trabecular meshwork and lens partitioning of corticosteroids: Implications for elevated intraocular pressure and cataracts. Arch. Ophthalmol..

[10] Chae J.B., Joe S.G., Yang S.J., Lee J.Y., Kim J.G., Yoon Y.H. (2012). An increase in intraocular pressure after intravitreal steroid injection facilitates reduction of macular edema. Eye.

[11] Kim K.T., Lee H., Kim J.Y., Lee S., Chae J.B., Kim D.Y. (2020). Long-Term Visual/Anatomic Outcome in Patients with Fovea-Involving Fibrovascular Pigment Epithelium Detachment Presenting Choroidal Neovascularization on Optical Coherence Tomography Angiography. J. Clin. Med..

[12] Rhee D.J., Peck R.E., Belmont J., Martidis A., Liu M., Chang J., Fontanarosa J., Moster M.R. (2006). Intraocular pressure alterations following intravitreal triamcinolone acetonide. Br. J. Ophthalmol..

[13] Shah A.R., Yonekawa Y., Todorich B., Van Laere L., Hussain R., Woodward M.A., Abbey A.M., Wolfe J.D. (2017). Prediction of Anti-VEGF Response in Diabetic Macular Edema After 1 Injection. J. Vitreoretinal Dis..

[14] Jones R., Rhee D.J. (2006). Corticosteroid-induced ocular hypertension and glaucoma: A brief review and update of the literature. Curr. Opin. Ophthalmol..

[15] Clark A.F., Wilson K., McCartney M.D., Miggans S.T., Kunkle M., Howe W. (1994). Glucocorticoid-induced formation of cross-linked actin networks in cultured human trabecular meshwork cells. Investig. Ophthalmol. Vis. Sci..

[16] Wilson K., McCartney M.D., Miggans S.T., Clark A.F. (1993). Dexamethasone induced ultrastructural changes in cultured human trabecular meshwork cells. Curr. Eye Res..

[17] Weinreb R.N., Mitchell M.D., Polansky J.R. (1983). Prostaglandin production by human trabecular cells: In vitro inhibition by dexamethasone. Investig. Ophthalmol. Vis. Sci..

[18] Wordinger R.J., Clark A.F. (1999). Effects of glucocorticoids on the trabecular meshwork: Towards a better understanding of glaucoma. Prog. Retin. Eye Res..

[19] Van der Valk R., Schouten J.S., Webers C.A., Beckers H.J., van Amelsvoort L.G., Schouten H.J., Hendrikse F., Prins M.H. (2005). The impact of a nationwide introduction of new drugs and a treatment protocol for glaucoma on the number of glaucoma surgeries. J. Glaucoma.

[20] Neufeld A.H., Dueker D.K., Vegge T., Sears M.L. (1975). Adenosine 3′,5′-monophosphate increases the outflow of aqueous humor from the rabbit eye. Investig. Ophthalmol..

[21] Dinarello C.A. (1991). Interleukin-1 and interleukin-1 antagonism. Blood.

[22] Alvarado J.A., Alvarado R.G., Yeh R.F., Franse-Carman L., Marcellino G.R., Brownstein M.J. (2005). A new insight into the cellular regulation of aqueous outflow: How trabecular meshwork endothelial cells drive a mechanism that regulates the permeability of Schlemm’s canal endothelial cells. Br. J. Ophthalmol..

[23] Fini M.E., Schwartz S.G., Gao X., Jeong S., Patel N., Itakura T., Price M.O., Price F.W., Varma R., Stamer W.D. (2017). Steroid-induced ocular hypertension/glaucoma: Focus on pharmacogenomics and implications for precision medicine. Prog. Retin. Eye Res..

[24] Mastropasqua R., D’Aloisio R., Di Nicola M., Di Martino G., Lamolinara A., Di Antonio L., Tognetto D., Toto L. (2018). Relationship between aqueous humor cytokine level changes and retinal vascular changes after intravitreal aflibercept for diabetic macular edema. Sci. Rep..

[25] Oakley R.H., Cidlowski J.A. (2011). Cellular processing of the glucocorticoid receptor gene and protein: New mechanisms for generating tissue-specific actions of glucocorticoids. J. Biol. Chem..

[26] Chin E.K., Almeida D.R.P., Velez G., Xu K., Peraire M., Corbella M., Elshatory Y.M., Kwon Y.H., Gehrs K.M., Boldt H.C. (2017). Ocular hypertension after intravitreal dexamethasone (ozurdex) sustained-release implant. Retina.

[27] Zarranz-Ventura J., Sala-Puigdollers A., Velazquez-Villoria D., Figueras-Roca M., Copete S., Distefano L., Boixadera A., García-Arumi J., Adan A. (2019). Long-term probability of intraocular pressure elevation with the intravitreal dexamethasone implant in the real-world. PLoS ONE.

[28] Srinivasan R., Sharma U., George R., Raman R., Sharma T. (2019). Intraocular pressure changes after dexamethasone implant in patients with glaucoma and steroid responders. Retina.

[29] Goni F.J., Stalmans I., Denis P., Nordmann J.P., Taylor S., Diestelhorst M., Figueiredo A.R., Garway-Heath D.F. (2016). Elevated Intraocular Pressure After Intravitreal Steroid Injection in Diabetic Macular Edema: Monitoring and Management. Ophthalmol. Ther..

